# High Intensity Focused Ultrasound for Treatment of Bone Malignancies—20 Years of History

**DOI:** 10.3390/cancers15010108

**Published:** 2022-12-24

**Authors:** Sin Yuin Yeo, Grischa Bratke, Holger Grüll

**Affiliations:** 1 Institute of Diagnostic and Interventional Radiology, Faculty of Medicine and University Hospital of Cologne, University of Cologne, Kerpener Str. 62, 50937 Cologne, Germany; 2 Department of Chemistry, Faculty of Mathematics and Natural Sciences, University of Cologne, Greinstr. 6, 50939 Cologne, Germany

**Keywords:** HIFU, focused ultrasound, bone, bone metastasis, osteosarcoma, primary bone tumor, pain, MR-HIFU, MRgFUS, USgFUS

## Abstract

**Simple Summary:**

High Intensity Focused Ultrasound (HIFU) allows non-invasive ablation of tumors and denervation of painful bone lesions. For 20 years, this method has been used for pain palliation in patients suffering from bone metastases as well as primary tumors. HIFU has also been used for local treatment of bone tumors. This review article summarizes 20 years of literature’s findings, published on HIFU treatment of bone malignancies with respect to pain palliation, treatment efficacy and safety.

**Abstract:**

High Intensity Focused Ultrasound (HIFU) is the only non-invasive method for percutaneous thermal ablation of tissue, with treatments typically performed either under magnetic resonance imaging or ultrasound guidance. Since this method allows efficient heating of bony structures, it has found not only early use in treatment of bone pain, but also in local treatment of malignant bone tumors. This review of 20 years of published studies shows that HIFU is a very efficient method for rapid pain relief, can provide local tumor control and has a very patient-friendly safety profile.

## 1. Introduction

Most often, bone lesions are formed as a result of metastatic spread of a solid primary tumor located elsewhere [1]. A comparably lower number of bone lesions may also result from primary benign or malignant bone tumors [2]. Regardless of their origin, common symptoms are bone pain and impairment of movement affecting quality of life, or structural instability [3]. However, treatment and pain management strategies strongly differ for bone metastases, primary malignant and benign bone lesions. Patients suffering from bone metastases are typically in a palliative setting with often limited life expectancies, where rapid and durable pain relief to increase quality of life is the leading objective. Current treatment options comprise medication such as analgesics, chemotherapy, hormonal therapy, bisphosphonates or endoradiotherapy, or physical methods, such as radiotherapy or image-guided percutaneous ablation [4,5].

While pain is also associated with primary malignant bone tumors, tumor control is the leading objective, especially for management of local disease. Here, the treatment aim is complete tumor eradication without risking local recurrence. The gold standard is complete surgical resection; however, this is often either not possible or associated with mutilations, such as amputation of limbs. As patients may refuse surgery, seeking alternative treatments, less invasive methods such as thermal ablation are playing an increasing role in local tumor treatment. Benign tumors ask again for a different approach which is directed to manage symptoms rather than complete tumor destruction. Especially here, minimally invasive thermoablative techniques are keys that provide pain relief and do not cause many side effects [6].

Image-guided percutaneous ablation comprises chemical ablation procedures (e.g., injection of ethanol) and physical ablation techniques to induce local tissue necrosis either by cooling (cryoablation) or by heating using, e.g., radiofrequency (RF), microwave applicators (MW) or high intensity focused ultrasound (HIFU) applicators [5,6]. While placement of cryo, RF and MW applicators still requires minimal invasive interventions, HIFU is the only technique which allows completely non-invasive focal heating of tissue using an extracorporeal transducer. At the focal site, HIFU waves interact with tissue, thereby causing vibration of molecules, which leads to energy absorption and local heating [7]. Tissue ablation occurs when temperatures reach >56 °C in situ. HIFU can be performed under magnetic resonance imaging (MR-HIFU) or ultrasound guidance (US-HIFU). MR-HIFU offers excellent soft tissue contrast treatment images for treatment planning and assessment, as well as near-real-time temperature information, based on the proton resonance frequency shift (PRFS) thermometry method, to monitor treatment [8]. On the other hand, US-HIFU brings the advantage of real-time imaging, and treatment is conducted based on change in tissue echogenicity. To date, HIFU has been used to treat uterine fibroids, prostate cancer, liver cancer, pancreatic cancer, essential tremors, desmoid tumors, bone metastases for palliative care, malignant primary bone tumors and others in a routine manner, or in clinical research [9,10]. A comprehensive list of clinical applications, and the state of research and regulatory approvals can be found in the latest State of the Field Report, published by the Focused Ultrasound Surgery Foundation [11].

**Figure 1:**
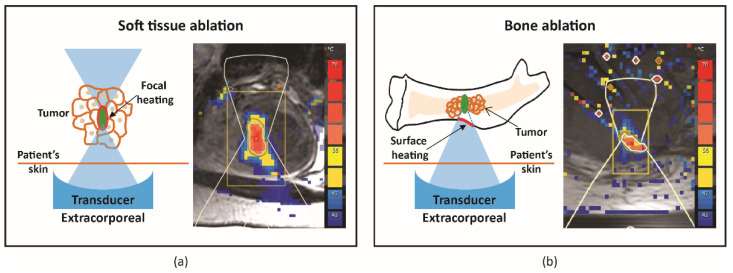
Schematic illustration and representative MR thermometry of uterine fibroid, soft tissue (**a**) and bone metastasis (**b**) ablation.

In soft tissue ablation, ultrasound energy is focused on a tight spot, leading to focal temperature increase when MRI is used for guidance, ablation, and tissue necrosis (Figure 1a). However, bone ablation works differently, as bone absorbs approximately 50 times more acoustic energy compared with soft tissue. Consequently, ultrasound energy accumulates at the intersection between the bone and ultrasound beam path, resulting in surface heating and ablation of the adjacent tissue (Figure 1b). There are a few challenges in bone ablation. For instance, bone has different acoustic properties compared with soft tissue. Hence, there is minimal penetration depth of US energy into the bone [12]. Additionally, there is no near-real-time MR thermometry nor change in echogenicity in the cortical bone for monitoring of treatment. As a result, treatment monitoring during bone ablation relies on the temperature or tissue change in the soft tissue adjacent to the bone. Based on this knowledge, strategies can be defined and adjusted based on the treatment aims and bone diseases.

**Figure 2:**
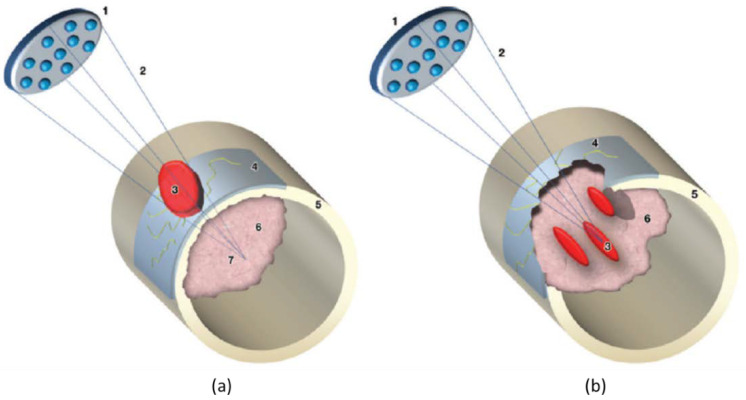
Diagrams illustrate the wide-beam approach used for treating bone metastases. 1 = transducer, 2 = focused ultrasound beam, 3 = area of ablation, 4 = invaded periosteum, 5 = cortical bone, 6 = metastasis, 7 = acoustic focus. When cortical bone is intact, as in (**a**) the ultrasound beam is entirely absorbed by the cortex surface, even if the focus of the ultrasound beam is placed beyond the cortical line. When a bone metastasis features transcortical growth (osteolytic lesion), as in (**b**) discontinuity of the bone surface is determined and the metastatic lesion can be treated with focal spots (**b**), as in MR imaging–guided focused ultrasound treatment of a regular solid lesion. Figures are reproduced with permission from Napoli A, Anzidei M, Marincola B C et al. MR Imaging–guided Focused Ultrasound for Treatment of Bone Metastasis ^1^. RadioGraphics 2013, 33, 1555–1568 [14].

Generally, HIFU is suitable for patients with localized (painful) non-spinal (apart from the posterior elements below the level of the conus medullaris) or non-skull lesions, which can be identified on MRI, CT, or US. The lesions should be ≥1 cm from skin and major nerve bundles, and should not require surgical stabilization (Mirel’s fracture risk score ≤7). Moreover, it is essential to ensure the absence of non-targeted bone, extensive scarring and/or hollow viscera within the acoustic beam path to allow clear acoustic access from the transducer to the target lesions for treatment [13,14,15]. The workflow on the day of HIFU treatment includes preparation of a patient with the required anesthesia and/or analgesic according to local hospital guidelines, positioning of the patient with the intended treatment region centered on the treatment window, localization of the target disease area with MRI or US imaging, treatment planning, treatment delivery, and post-treatment assessment and care [14]. For pain palliation of bone metastasis, in the presence of cortical bone, the treatment focus spot can be positioned behind the cortical bone, i.e., in the intramedullary space (wide-beam approach, Figure 2a) or on the surface of the cortical bone (Figure 2b). Both approaches aim at ablation of the richly innervated periosteum to provide pain relief, with the earlier strategy relying on the US energy which accumulates at the bone/US beam intersection, while the latter strategy utilizes the energy at the focal spot (direct approach). For treatment of bone metastasis or primary malignant tumor with compromised cortical bone and aiming for pain palliation and/or tumor control, the treatment focus spot can be placed directly on the tumor tissue as well as the bone/tumor tissue interface (Figure 2b). More detailed information on patient selection, clinical indications, technical consideration and a step-by-step guide for performing MR-HIFU can be found in [13,14].

This review aims to take the readers on a 20-year journey of HIFU treatment of bone metastasis and primary malignant bone tumors, focusing on treatment effects and safety.

## 2. Methods

A PubMed literature search was conducted for HIFU treatments of bone metastasis and primary malignant bone tumors articles using the following search terms: (“Bone Metastases” OR “bone mets” OR “bone metastasis”) AND (“focused ultrasound” OR HIFU), (“focused ultrasound” OR “HIFU”) and (“bone tumor” OR “bone tumour”), and (“focused ultrasound” OR “HIFU”) and (“bone tumor”). The titles and abstracts were screened for presence of efficacy and safety results, and included in this review. Thereafter, the references of the identified articles were checked for additional related articles. Based on our criteria, a total of 32 and 7 publications were identified for bone metastasis and primary malignant bone tumor, respectively. The number of publications per year for bone metastasis is 1 in 2007, 1 in 2008, 1 in 2009, 1 in 2010, 1 in 2011, 1 in 2013, 2 in 2014, 2 in 2015, 2 in 2016, 3 in 2017, 5 in 2018, 5 in 2019, 1 in 2020, 3 in 2021, and 3 in 2022. The number of publications per year for primary malignant bone tumor is 1 in 2002, 1 in 2009, 2 in 2010, 1 in 2013, 1 in 2017, and 1 in 2019.

## 3. Results

### 3.1. Treatment of Bone Metastasis

HIFU can be used to treat osteoblastic, osteolytic and mixed bone lesions, and was first applied with the aim to provide pain palliation for radiation therapy (RT)-refractory bone metastasis patients. In 2007, Catane et al. treated 13 patients who had exhausted all treatment options with MR-HIFU. Preliminary results showed that at a mean follow-up of 59 days, the visual analogue scale (VAS) pain score improved. The onset of these improvements was 3 days following treatment. Besides transient post-procedural pain, which resolved within 3 days after treatment, there were no severe adverse events [16]. The feasibility and efficacy of MR-HIFU to provide pain relief were subsequently shown in 11 patients, where VAS scores significantly decreased from an average of 6.0 before treatment to 0.5 at 3 months after treatment (*p* value < 0.01), with a 100% response rate [17]. In a randomized, placebo-controlled phase 3 trial, the response rate, defined as at least a 2-point decrease in the worst numerical rating scale (NRS) pain score and no more than a 25% increase in morphine equivalent daily dose (MEDD) intake from baseline to 3 months post treatment, was 64.3% in the MR-HIFU arm versus 20.0% in the placebo arm (*p* value < 0.001) [15]. Additionally, MR-HIFU has also been used to treat metastatic bone tumors in children with an average age of 4.27 ± 0.83 years old. In a 3-month follow-up period, NRS and VAS pain scores significant decreased from 6.27 ± 1.53 to 2.18 ± 1.04 and from 6.56 ± 2.38 to 1.85 ± 0.96, respectively [18]. A systemic review and meta-analysis, including 15 studies with 362 patients, reported the average pain score at baseline was 6.74 (95% CI: 6.30–7.18), and decreased to 4.15 (95% CI: 3.31–4.99) at 0–1 week, 3.09 (95% CI: 2.46–3.72) at 1–5 weeks, and 2.28 (95% CI: 1.37–3.19) at 5–14 weeks [19]. In a separate systemic review and meta-analysis including 33 studies and 1082 patients, pain response was observed in 79% of patients at 3 months after HIFU treatments, with a mean pain score difference of −3.8 and −4.4 between baseline and 1-month and 3-month follow-up, respectively [20]. While most studies have a short-term follow-up for up to 3 months due to the palliative nature of the treatments, a few studies reported long-term durability of HIFU. A case report on treatment of pelvic bone metastasis showed a patient with a VAS pain score of 9 prior to the MR-HIFU treatment, which reduced to 2 after the treatment and remained stable for one year [21]. In a retrospective study enrolling 26 patients receiving US-HIFU treatments, the mean VAS pain scores changed from 6.69 ± 1.44 at baseline to 5.96 ± 1.14 at 12 months post treatments. Albeit a 10.9% reduction in pain score, the difference was significant (*p* value < 0.01) [22]. The 1-, 2-, 3-, and 5-year survival rates of 12 patients undergoing US-HIFU treatments were 83.3%, 16.7%, 0%, and 0%, respectively [23].

Concurrent with pain palliation, patients who underwent HIFU treatments also experienced improvement in quality of life (QoL) and reduction in analgesic intake. QoL has been assessed using different questionnaires, such as the brief pain inventory (BPI) QoL [15,20,24,25], the European Organization for Research and Treatment of Cancer (EORTC)’s Quality of Life Questionnaire Core 15 Palliative Care (QLQ-C15-PAL) [26,27], and the EORTC QoL Questionnaires for Patients with Bone Metastases 22 (EORTC QLQ-BM22) [22,26,28], or EORTC QLQ-C30 [18]. As presented by Hurwitz et al. in a randomized controlled trial (RCT), the BPI-QoL score at 3 months after MR-HIFU therapy was 2.4 points better than the placebo arm [15]. At the same 3-month time point, the results from a meta-analysis showed a reduction in BPI-QoL scores from 36.2 to 28.5 [20]. When assessed using the QLQ-C15-PAL questionnaires, MR-HIFU improved the median scores for health status at 30 days post therapy, particularly for physical (baseline: 40; 30 days follow-up: 73.3) and emotional function scores (baseline: 66.7; 30 days follow-up: 100) [27]. The overall QoL of MR-HIFU-treated patients, evaluated using the QLQ-C15-PAL questionnaires, improved from 49.99 ± 22.04 to 56.07 ± 27.14 at 90 days after treatments [26]. In a 23-patient study, MR-HIFU significantly reduced the QLQ-BM22 scores at 1-week (44 ± 12), 1-month (42 ± 12), and 3-month (39 ± 12) follow-up, compared with scores before treatments (52 ± 13) [28]. In a 12-month follow-up study, functional interference scores showed a decreasing trend, starting from 2 months (69.83 ± 4.67) until 6 months (66.59 ± 3.93) after treatment, compared with baseline (89.66 ± 6.54). From 8 months after treatments, scores started increasing until 72.84 ± 5.13 at 12 months after treatment, but remained significantly different with respect to baseline. Similarly, psychosocial aspect scores significantly decreased from baseline (90.87 ± 4.25) to 2 months (59.29 ± 11.86) after treatments. However, the scores started increasing until 72.59 ± 12.92 (*p* value < 0.01) at the 12-month follow-up timepoint. These results suggested that while US-HIFU significantly improved the functional and psychosocial wellbeing of patients throughout the 12-month follow-up period, there was room for improvement in terms of durability at 12 months after treatment [22]. The analgesic consumption has been reported as morphine equivalent daily dose (MEDD). The median MEDD prior to MR-HIFU treatment was 37.5 mg and reduced to 14.3 and 7.3 mg at 7 and 30 days after treatment, respectively. At 90 days post treatment, there was no further increase in MEDD [27]. Based on 2 separate meta-analyses, the change in MEDD after treatments was −15.11 (2 weeks), −10.87 (1 month), and −5.53 (3 months) [19]; meanwhile, on average, 55.8% of patients were able to stop taking pain medication and 33% of patients were able to reduce their analgesic intake [20]. Reducing the need for pain medication would reduce analgesia-related side effects and improve the wellbeing and QoL of patients in palliative settings.

**Figure 3:**
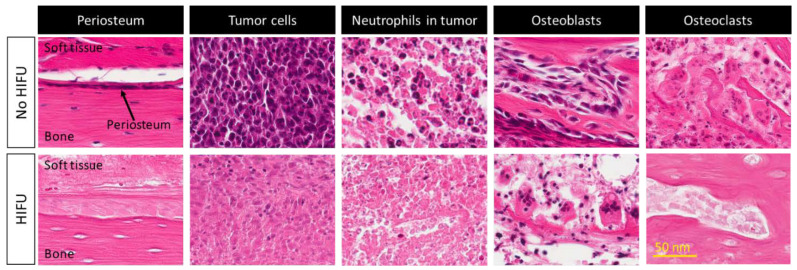
Histological images (hematoxylin and eosin) of viable and necrotic periosteum, tumor cells, neutrophils in tumor microenvironment, osteoblasts, and osteoclasts before and after MR-HIFU treatment, respectively, in a preclinical osteoblastic rat model. Figures adapted and reprinted from Bone, Volume 81, Yeo SY, Elevelt A, Donato K, van Rietbergen B, ter Hoeve ND, van Diest PJ, and Grüll H, Bone Metastasis Treatment Using Magnetic Resonance-guided High Intensity Focused Ultrasound, 513–523, 2015, with permission from Elsevier (Amsterdam, The Netherlands) [29].

**Figure 4:**
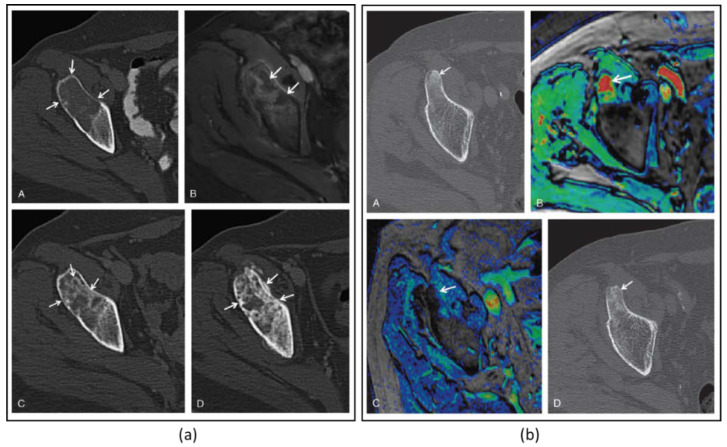
(**a**) Images of a 64-year-old woman with iliac bone metastasis from breast cancer. A: Axial CT image shows a wide lytic lesion located in the right anterior superior iliac spine (arrows) with evidence of focal cortical erosion, causing severe pain (pain severity score, 10). B: Axial contrast enhanced T1-weighted MRI (CE-MRI) after the MR-HIFU treatment shows the presence of some small areas of NPV (arrowheads) inside the lesion and at the periosteal margin. C: At the 2-month follow-up, axial CT identified the presence of some focal areas of de novo mineralization inside the treated tissue with partial restoration of cortical borders (arrowheads). D: At 3 months after the treatment, the lesion showed further denovo remineralization of the ablated tissue (arrowheads). On the basis of MDA criteria, this patient was classified as a partial responder; pain severity score dropped to 0 throughout the follow-up period. (**b**) Axial CT image of a 63-year-old man with primary prostate cancer. A: The arrowhead shows a metastasis located in the right anterior-superior iliac spine with a hyperdense pathologic nodular nucleus. B: The color-coded T1-weighted CE-MRI acquired reveals intense enhancement of the pathologic area (arrow). At 3 months after the MR-HIFU treatment, CE-MRI (C) showed a marked reduction of the lesion enhancement (arrow). D: The axial CT image acquired the same day reveals increased density in the treated area and the disappearance of the nodular pathologic tissue (arrow). On the basis of the MDA criteria, this patient was classified as a complete responder. Figures adapted and reprinted with permission from Napoli A, Anzidei M, Marincola BC, Brachettti G, Ciolina F, Cartocci G, Marsecano C, Zaccagna F, Marchetti L, Cortesi E, and Catalano C, Primary Pain Palliation and Local Tumor Control in Bone Metastases Treated With Magnetic Resonance-Guided Focused Ultrasound, Investigative Radiology, 48, 6, 351-358, https://journals.lww.com/investigativeradiology/Abstract/2013/06000/Primary_Pain_Palliation_and_Local_Tumor_Control_in.1.aspx accessed on 21 December 2022 [30].

**Figure 5:**
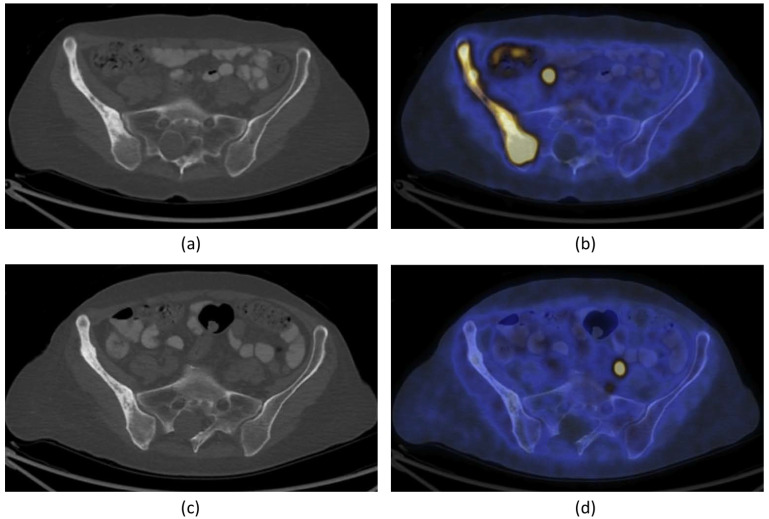
A 66-year-old woman with bone metastasis in the right ilium. Trans-axial ^18^F-FDG PET/CT slide at the level of the pelvis (**a**): CT; (**b**): fused PET/CT) demonstrates pathological increase in ^18^F-FDG uptake in a larger mixed lytic-sclerotic lesion before treatment. After MR-HIFU treatment, no ^18^F-FDG uptake was observed in the right ilium (**d**). There is, however, no change in the bony structure on CT (**c**). The findings are consistent with healed metastasis in the right ilium with no evidence of active residual malignancy in the site. FDG = fluorodeoxyglucose; PET = positron emission tomography; CT = computed tomography). Figures adapted and reprinted from Journal of Pain and Symptom Management, Volume 56 Issue 1, Eisenberg E, Shay L, Keidar Z, Amit A, and Militiau D, Magnetic Resonance–Guided Focused Ultrasound Surgery for Bone Metastasis: From Pain Palliation to Biological Ablation?, 158–162, 2018, with permission from Elsevier (Amsterdam, The Netherlands) [32].

It was hypothesized that one of mechanisms of pain relief was ablation of the periosteal innervation causing a fast and enduring pain palliation. To address the above question, a detailed preclinical MR-HIFU study has been conducted in rats suffering from a painful prostate bone metastasis within the femur. Multimodal imaging and histological analysis demonstrated that pain control obtained after HIFU ablation was indeed associated with denervation of the periosteum (Figure 3) [29]. Besides managing pain, the same study demonstrated that HIFU provides local bone lesion control, where necrosis of tumor cells, inflammatory cells, osteoblasts, and osteoclasts were evident after MR-HIFU ablation (Figure 3) [29]. These preclinical results are consistent with outcome observed in multiple clinical studies [17,23,27,30,31]. Contrast-enhanced T1-weighted MR images showed an average decrease in enhancing tumor volume of 50.2% at 1 month after treatment compared with baseline, and 44% at 3-month compared with 1-month follow-up after MR-HIFU treatments. Additionally, in 56% of patients, CT images at 3 months revealed a bone density increase [17]. Napoli et al. evaluated tumor control in 18 patients using the MD Anderson criteria [30]. Results showed that at 3 months after MR-HIFU treatments, 11.1% of patients experienced complete response (CR), 22.2% of patients had partial response (PR), 55.6% of patients had stable disease (SD), and 11.1% patients had progressive disease (PD). CT images illustrated a bone density increase in combination with restoration of the cortical bone in 27.7% of patients with osteolytic lesions, a bone density decrease in 16.7% of patients with sclerotic lesions and no bone density changes in 55.6% of patients. Figure 4 shows examples of bone normalization after MR-HIFU treatments [30]. In a separate study, radiological response according to the MD Anderson criteria was observed in 9 out of 11 patients [27]. In addition, the World Health Organization (WHO) standard has also been used to investigate tumor response; in a study where 12 patients were treated with US-HIFU, the overall response rate was 75% where CR, PR, moderate response, SD, and PD were 41.7%, 43.3%, 8.3%, 8.3% and 8.3%, respectively [23]. Positron emission tomography (PET)/CT in combination with ^18^F-fluorodeoxiglucose (^18^F-FDG) is another imaging modality used to assess tumor metabolic response. At 4–6 weeks after US-HIFU, no abnormal accumulation of activity at tumor sites [23]. In a patient with mixed lytic-sclerotic lesion at the iliac bone, no FDG uptake was observed at 3 months after MR-HIFU treatment (Figure 5) [32]; meanwhile, in a separate case study involving an osteolytic lesion at the iliac bone, a 35.1% decrease in standard uptake value (SUV) was noted at 3 months after MR-HIFU treatment [21], confirming tumor response in both cases. Using the PET response criteria in solid tumors (PERCIST 1.0), another study reported PR in 5 out of 12 patients and SD in 2 out of 12 patients; 4 out of 12 patients had PD at a median follow-up of 90 days after MR-HIFU [27].

Researchers have also investigated changes in urinary cytokines or biomarkers. The urine cytokines, such as interleukin 1β (IL-1β), IL-4, IL-6, IL-10, tumor necrosis factor α (TNF-α) and tumor growth factor α (TGFα), were measured 3 days before and 2 days after MR-HIFU treatment. All urine cytokines showed no significant differences between the two time points, with the exception of TGFα demonstrating an overall decrease [33]. Furthermore, pro-inflammatory cytokines and anti-inflammatory cytokines were measured in 10 patients undergoing MR-HIFU treatments. It was noted that eotaxin, GRO, IL-8, IL-13, IP-10, MCP-1, MIP-1β, RANTES and SIL-2Rα significantly decreased at 2 days after treatment. For patients experiencing positive pain response, GRO, IFN-γ, IP-10, IL-13, MCP-1, and RANTES significantly decrease after MR-HIFU [34]. Bone turnover markers, such as CTX and RANK-L, decreased at 1 and 3 months after MR-HIFU treatment. Generally, both markers were lower in patients exhibiting response compared with patients showing SD or PD. However, the changes were not significant, and there was no correlation to pain or tumor response [27]. A few-shot machine learning approach was proposed as a prognosis prediction model for pain palliation for patients treated with MR-HIFU. This model took into consideration Karnofsky performance status (KPS), IL-6, INFγ, TNFα and vascular endothelial growth factor (VEGF) levels and had an accuracy of 95%, an area under the curve (AUC) of 0.95 and Akaike information criteria (AIC) of 17.14 [35].

To date, results have shown that HIFU was not only effective for pain relief, improvement of QoL, reducing analgesic consumption, and tumor and bone lesion control, but also safe for the treatment of bone metastases. Several studies have reported the absence of severe adverse events following HIFU treatments [17,22,24,26,30,33,36,37]. In the RCT where 112 patients were treated with MR-HIFU, 63 adverse events (AEs) were reported, with 60.3% of them being transient and resolving on the treatment day, and 14.3% resolving within 1 week after treatment. The most common AE was pain experienced during sonication, followed by positioning pain and postprocedural pain. One patient had a third-degree skin burn due to noncompliance with treatment guidelines, which resolved within 2 months after treatment. Fracture was observed in 2 patients, but one of the fractures was outside the treatment area. At 3 months follow-up, 4 AEs did not resolve (fracture outside treatment area, mild skin numbness, hip flexor neuropathy and fatigue) [15]. A meta-analysis evaluating adverse events according to the common terminology criteria for adverse events (CTCAE) grading, included 799 patients from 26 studies, showed that 5.9% of patients had AEs with CTCAE grade ≤2, while 0.9% of patients had AEs with CTCAE grade ≥3 [20].

HIFU has also been compared with conventional RT in a matched-pair study [38]. Here, MR-HIFU was shown to provide pain relief within 1 week after treatment, which is significantly faster compared with RT. At 1 and 2 months after treatment, there was no significant difference between the pain scores for patients who received HIFU or RT. Similarly, the treatment response rates were significantly higher in patients receiving MR-HIFU at 1 and 2 weeks after treatment, while at 1,2 and 3 months after treatments, there were no significant differences between the two groups. At all follow-up time points within 3 months, the MEDD did not significantly differ between HIFU and RT groups. In terms of AEs, 2 patients in the MR-HIFU group reported pain during treatment, resulting in temporary interruption of the treatment procedures, and 1 case of myositis which resolved within 2 weeks after treatment. On the other hand, 2 patients in the RT group experienced diarrhea and were treated with antidiarrheal medication, leading to stabilization of the AE. Although HIFU has been utilized to date as an alternative to RT, future studies should assess the synergistic effects of HIFU, and RT. Bartels et al. recently showed that it was feasible and safe to combine both MR-HIFU and RT in 6 patients. From a logistical perspective, MR-HIFU treatments were performed within 4 days after RT. The onset of pain relief was at 3 days after treatment, with 83% of patients experiencing pain response at 7 days after treatment and 60% of patients experiencing pain response at 4 weeks after treatment. No treatment-related severe AEs were noted [39]. A European Union-funded multicenter three-arm, randomized trial (FURTHER) to compare MR-HIFU, RT and a combination treatment of RT followed by MR-HIFU for first line treatment of painful bone metastases is currently ongoing (ClinicalTrials.gov Identifier: NCT04307914; https://projectfurther.org (accessed on 3 December 2022)). Table 1 summarizes the publications on HIFU treatment of bone metastasis with their respective clinical outcomes and adverse events.

### 3.2. Treatment of Primary Malignant Tumor

Primary bone cancers comprise osteosarcoma, Ewing sarcoma and chondrosarcoma, all accounting for less than 1% of diagnosed cancers per year [2]. In particular, children and young adults are often diagnosed with osteosarcomas, which have a peak incidence at the ages of 10 to 14 years. Though they can occur in principle in all bones, most commonly they are found in the distal femur. Management of bone lesions depends on tumor characteristics and particularly the tumor stage, with surgery as standard care, in combination with chemotherapy and/or radiotherapy if needed. Besides RF and MWV ablation, HIFU has been used in numerous clinical studies for the treatment of malignant bone lesions. While HIFU treatment of bone metastases aims at rapid and long-lasting pain palliation, treatment of primary malignant bone lesions requires local tumor control, especially once performed with curative attempts in patients with local disease. Consequently, most studies assessed local control using imaging protocols, in addition to survival and, in few cases, recurrences. Assessment of pain reduction and quality of life received less attention compared with HIFU treatments of painful bone metastases.

**Figure 6:**
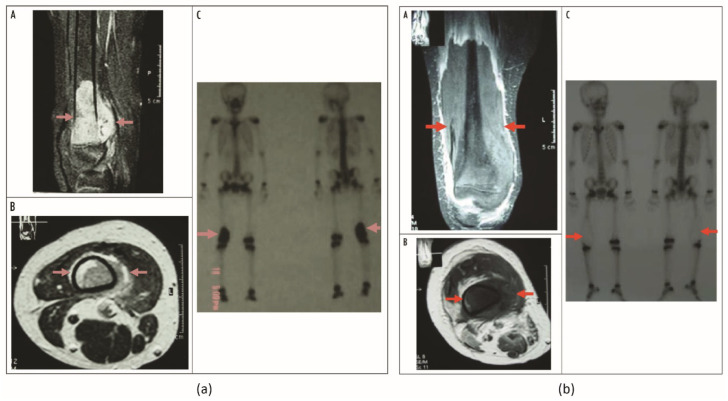
(**a**) Osteosarcoma in inferior segment of right femoral bone after neoadjuvant chemotherapy before HIFU. DCE-MR images obtained from (A) coronal plane and (B) transverse plane show abundant blood supply and survival tumor in the osteosarcoma lesion (red arrow). (C) Static bone imaging of ^99m^Tc-MDP, with radioactivity concentration in inferior segment of right femoral bone (red arrow); tumor survived. (**b**) The same patient 8 weeks after 2 HIFU treatments. DCE-MR images obtained in (A) coronal plane and (B) transverse plane show no evidence of contrast enhancement in the treated lesion (red arrow), with a papery enhanced band on the verge of inactivated area, which is indicative of complete coagulation necrosis in the osteosarcoma. (C) Static bone imaging of ^99m^Tc-MDP, with radioactivity concentration disappeared in inferior segment of right femoral bone (red arrow); tumor is inactivated. There is a reaction band along with the verge of inactivated area of tumor with higher radioactive uptake. Figures adapted from Osteosarcoma: Limb Salvaging Treatment by Ultrasonographically Guided High-intensity Focused Ultrasound, Li C, Wu P, Zhang L, Fan W, Huang J and Zhang F, Cancer Biology & Therapy, 15 June 2009, and reprinted by permission from Taylor & Francis Ltd., http://www.tandfonline.com (accessed on 7 December 2022) [41].

The use of HIFU to treat primary malignant tumors was first published in 2002 by Chen et al. wherein 44 patients were treated with US-HIFU. At 7–14 days after treatment, SPECT images showed absence of abnormal ^99m^Tc-MDP signal, indicating a radioactive “cold” area. Starting from 3 months after the treatment, revascularization of the treated region was observed. At 1 year after the treatment, revascularization and bone remineralization of the whole treated bone area were noted. The local recurrence rate was 9.1%. Within the follow-up period, the average Enneking score was 21.5 points for 81.8% of patients, suggesting that most patients were able to lead a normal life. The overall survival rate was 84.1%, with 68.2% of patients having tumor-free survival and 15.9% of patients surviving with tumors. A total of 8 complications (2 secondary infections, 3 pathological fractures, 1 epiphyseal separation and 2 common peroneal nerve injuries) were reported, and with the exception of 1 secondary infection case requiring amputation, all complications improved with treatments [40]. Li et al. evaluated HIFU in the salvage treatment of 7 patients diagnosed with extremity osteosarcomas mostly located in the distal femur [41]. Tumor control was evaluated using MRI and SPECT imaging at 1 and 3 months after HIFU intervention, which was performed using diagnostic ultrasound for image guidance. For SPECT imaging, the tracer ^99m^Tc-MDP was used to image bone tumor-related activity before and after treatment. Complete response (42.9%) was defined as no ^99m^Tc-MDP uptake in ablated tumors at 1–3 months after HIFU, i.e., complete tumor ablation (“cold” lesion); meanwhile tumors with PR (42.9%) typically showed uptake along the outer rim of the tumor (Figure 6). One patient showed progressive disease and developed pulmonary metastases. Median survival was 68 months with a 5-year survival of 71.4%. Treatment-related complications, such as local edema, first- and second-degree skin burns and limb paresthesia all resolved within weeks after treatment. A second study by Li et al. assessed the US-HIFU treatment of 25 patients, comprising 13 patients with primary osteosarcomas and another 12 patients with bone metastases [23]. The follow-up protocol comprised multimodal imaging with MRI, PET/CT, SPECT (^99m^Tc-MDP) and CT performed 4–6 weeks after HIFU treatment to assess tumor ablation and response. Complete tumor ablation, i.e., the entire tumor being necrotic or disappeared, was observed in 6 cases (46.2%), partial response was observed in 5 patients (38.5%) and moderate response in 1 patient (7.8%). One patient showed progressive disease (7.8%). Survival at 1, 2, 3, and 5 years was 100.0%, 84.6%, 69.2% and 38.5%, respectively. An average reduction of pain was observed after treatment from 1.85 ± 0.69 to 0.12 ± 0.33 (VRS scale). Similarly to earlier studies, most treatment-related side effects resolved within 2 weeks. However, peripheral nerve damage was reported in patients in which the nerve was located less than 1 cm distance from the ablated area. In general, 1 cm is considered an acceptable safety margin, and target lesions that are less than 1 cm from nerve bundles have been excluded for treatment in prior studies [14,15]. Two publications by Li et al. also examine blood samples before and after treatment. Levels of alkaline phosphatase and lactic acid dehydrogenase before and directly after treatment were comparable, but reduced after 1 and 2 months after treatment [23,41]. Chen et al. presented a study with 80 patients diagnosed with primary lesion from 62 osteosarcomas, 1 periosteal osteosarcoma, 1 periosteal sarcoma, 3 Ewing sarcomas, 10 chondrosarcomas and 3 others [42]. Clinical treatment for osteosarcoma and Ewing sarcoma patients comprised neoadjuvant and adjuvant chemotherapy interleaving the HIFU treatment, while all other patients received HIFU ablation only. Most tumors were again located in the distal femur (41%). CT, MRI, and SPECT (^99m^Tc-MDP) were used to assess tumor response. At 14 days after US-HIFU treatments, CR (100% tumor ablation) was observed in 69 patients (86%), while PR (>50% tumor ablation) was achieved in all remaining 11 patients where tumor location and size prohibited complete ablation. Interestingly, only 5 patients out of the 69 patients with completely ablated tumors showed a local tumor progression after 36.8 ± 22.9 months. An important finding of this study was that the patient subgroup (stage IIb, HIFU + chemotherapy) who complete the full treatment schedule of complete tumor ablation and all cycles of chemotherapy have a significantly longer survival compared with patients with either incomplete tumor ablation (PR) or chemotherapy cycles. Besides typical HIFU-related side effects that resolved within two weeks after treatment, bone fractures were also reported for 6 patients. However, it remains unclear if these fractures can be linked to the HIFU treatment as such, as they frequently occur in this patient population due to the poor structural integrity of the bone [42]. Wang et al. performed a retrospective analysis of 11 patients (4 osteosarcomas, 2 Ewing sarcomas, 1 chondrosarcoma, 1 neuroectodermal and 1 giant cell tumor) treated with US-HIFU [43]. MR imaging was performed to assess tumor ablation and response. Four patients received HIFU treatment with a curative intent, i.e., achieving 100% tumor ablation. One of these patients showed a local recurrence which was successfully retreated. At the time of publication, all patients treated with curative intent were alive, ranging from 11–154 months. In all other cases, treatment was performed in a palliative setting with partial ablation of 79.1 ± 8.7% tumor volume. These patients were lost due to progression and metastatic disease during follow up. Six patients also reported mild to moderate pain requiring oral analgesics for pain relief. Pain resolved after treatment and patients were reported pain free without additional medication. Singh et al. reported a retrospective study of 16 primary sarcomas treated using MR-HIFU [44]. For primary lesions, a treat-and-resect study was performed with surgery scheduled 14 days after HIFU. Specimens were histologically examined for ablation. Consequently, pain scores could not be assessed and nor could long term survival related to HIFU treatment. Histological analysis revealed 100% tissue necrosis in tumor areas treated with MR-HIFU. However, as it remains unclear how the treated tumor volume relates to the total tumor volume, the statement of 100% tissue necrosis in treated areas is difficult to use as a measure for efficacy. Additionally, this study reports typical HIFU-related side effects that all resolve within days after treatment.

To our knowledge, the only prospective two-arm randomized study, which compares chemotherapy (adriamycin) combined with HIFU (observation group, OG, *n* = 36) with chemotherapy alone (control group, CG, *n* = 36) for treatment of osteosarcomas was presented by Chunqiu Wang [45]. Assessment of efficacy, classified as CR, PR, SD, PD, was performed using the RECIST criteria of the WHO. For the OG, the overall response (88.9%) and disease control rate (94.4%) were found to be significantly higher compared with the CG (66.7% and 75.0%, respectively). While the survival did not differ during the first year of follow up, survival rates at 2 (69.4%) and 3 years (38.9%) were significantly higher in the OG compared with control (2y: 52.8%; 3 y: 22.2%), indicating a survival benefit for patients receiving the combined treatment protocol. Adverse events were related to chemotherapy and did not differ for OG and CG suggesting that HIFU does not increase chemo-related side effects nor adds extra side effects. Adjacent analysis of blood markers indicated an increase of TNF-α, IL-2 and IL-8, which was higher for the OG compared with CG. As TNF-α and ILs are cytokines that can strengthen the immune response, a protocol combining adriamycin with HIFU may be able to enhance the therapeutic outcome via activation of the immune system. Table 2 summarizes the publications on HIFU treatment of primary malignant bone tumors with their respective clinical outcomes and adverse events.

**Table 1:** A summary of clinical studies on HIFU treatment of bone metastasis.

Study	Patient Number	Imaging Guidance	Lesion location	Pain Assessment ^a^	Tumor Response ^b^ and Survival	Pain Medication or MEDD ^c^	Quality of Life ^d^	Adverse Events
Catane et al. 2007 [16] Retrospective study	13	MRI	Ilium: 10 Ischium: 1 Sacrum: 1 Humerus: 1 Femur: 1	Score: Center 1: Pre: 5.5 3 d: 2.3 2 wk: 1.2 1 m: 0.5 3 m: 0.3 Center 2: Pre: 5.4 3 d: 3.9 2 wk: 4.5 1 m: 2.8 3 m: 2.0 6 m: 1.0	n/a	Improvement in pain relieve medication.	n/a	Transient post procedural pain: 1, resolved within 3 d. No SAE
Gianfelice et al. 2008 [17] Prospective study	11	MRI	Ilium: 10 Scapula: 2 Ischium: 1 Clavicula: 1	Score: Pre: 6.0 3 d: 3.7 2 wk: 2.2 1 m: 1.3 3 m: 0.5	Tumor response (CE-T1w MRI): An average decrease of enhancing tumor volume by 50.2% at 1 m after HIFU compared with baseline, and 44% at 3 m compared with 1 m follow-up after HIFU.	Medication: Discontinued: 63.6% Reduction ≥ 50%: 36.4%	n/a	None reported
Liberman et al. 2009 [36] Prospective study	31	MRI	Ilium: 18 Ischium: 4 Sacrum: 4 Scapula: 2 Femur: 1 Humerus: 1 Clavicula: 1	Score: Pre: 5.9 (3.5–8.5) 3 d: 3.8 (0–8.5) 3 m: 1.8 (0–8) Response (IBMCWP): CR: 36% PR: 36%	n/a	Opioid-based medication (*n* = 12): Reduction: 67% Increase: 22% Non-opioid medication (*n* = 13): Reduction: 100%	n/a	None reported
Li et al. 2010 [23] Prospective study	12	US	Ilium: 5 Rib: 6 Sternum: 1	Score (verbal rating scale): Pre: 1.75 ± 0.97 Post: 0.17 ± 0.39 Response: CR: 87.5%	Tumor response at 4–6 wk (WHO standard): CR: 41.7% PR: 33.3% moderate response: 8.3% SD: 8.3% PD: 8.3%	n/a	n/a	Data combined with primary malignant bone tumor. 1^st^-degree skin burn: 12, resolved within 2 wk. 2^nd^-degree skin burn: 2, resolved after 4 wk. Lack of limb sensation during HIFU: 3, resolved after HIFU has completed.
Candiano et al. 2011 [21] Case report	1	MRI	Ilium	Score: Pre: 9 12 m: 2	Tumor response at 3 m (^18^F-FDG PET/CT): 35.1% reduction in SUV.	No analgesic changes.	Improvement in QoL.	n/a
Napoli et al. 2013 [30] Prospective study	18	MRI	Ilium: 10 Scapula: 3 Extremities: 4 T7 vertebra: 1	Score: Pre: 7.1 ± 2.1 1 m: 2.5 ± 1.4 3 m: 1.0 ± 1.1 Response at 3 m (IBMCWP): CR: 72.2% PR: 16.7% PD: 11.1%	Tumor response at 3 m (MDA criteria): CR: 11.1% PR: 22.2% SD: 55.6% PD: 11.1%	Medication at 3 m: Discontinued:72.2% Stable: 16.7% Reintroduction: 11.1%.	BPI-QoL: Pre: 4.8 ± 1.8 1 m: 1.8 ± 1.0 2 m: 0.7 ± 0.6 3 m: 0.5 ± 0.9	None reported
Huisman et al. 2014 [46] Observational cohort study	11	MRI	Sacrum: 2 Rib: 3 Pubic: 4 Femur: 2 Humerus: 1	Score: Pre: 8.1 ± 1.3 3 d: 6.6 ± 2.0 1 m: 2.3 ± 2.1 Response at 3d (IBMCWP): PR: 55% PD: 9% Response at 1 m (IBMCWP): CR: 11% PR: 56%	n/a	Medication at 1 m: Reduction: 22.2% Stable: 66.7% Increase: 11.1%	n/a	Post procedural pain: 1 1^st^-degree skin burn: 1 No SAE
Hurwirtz et al. 2014 [15] Randomized, placebo-controlled, single-blind, multicenter, pivotal trial	147 (112 HIFU; 35 placebo)	MRI	HIFU: Pelvis: 70 Sacrum and coccyx: 12 Rib and sternum: 16 Extremities: 7 Scapula: 7 Placebo: Pelvis: 19 Sacrum and coccyx: 6 Rib and sternum: 6 Extremities: 3 Scapula: 1	Score at 3 m: Mean reduction from baseline in worst NRS: HIFU: 3.6 ± 3.1 Placebo: 0.7 ± 2.4 Response at 3 m (IBMCWP): HIFU: 64.3% (CR: 23.2%) Placebo: 20.0% (CR: 5.7%)	n/a	Medication at 3 m: Discontinued: HIFU: 27% Placebo: 14% Reduction: HIFU: 17% Placebo: 0%	MR-HIFU was 2.4-point superior to placebo in BPI-QoL scores at 3 m follow-up.	HIFU: Any AE: 51 Sonication pain: 36 Position pain: 9 Post procedural pain: 5 Fatigue: 2 Neuropathy: 2 Fracture: 2 Skin burn: 2 Blood in urine: 1 Fever: 1 Myositis: 1 Numbness: 1 Skin rash: 1 60.3% resolved on HIFU treatment day and 14.3% resolved within 1 wk. Placebo: Any AE: 1 Position pain: 1
Gu et al. 2015 [28] Prospective study	23	MRI	The following locations were included during screening, but the number of patients treated per location was not specified. Ribs, extremities (including joints), pelvis, shoulder joints or third lumbar vertebrae and below of the posterior part of the spine.	Score: Pre: 6.0 ± 1.5 1 wk: 3.7 ± 1.7 1 m: 3.1 ± 2.0 3 m: 2.2 ± 1.0	n/a	n/a	BPI-QoL: Pre: 39 ± 16 1 wk: 27 ± 18 1 m: 26 ± 18 3 m: 21 ± 18 QLQ-BM22: Pre: 52 ± 13 1 wk: 44 ± 12 1 m: 42 ± 12 3 m: 39 ± 12	Pain in therapy area: 3, resolved within 1 wk. Numbness in lower limb: 1, resolved after physiotherapy.
Joo et al. 2015 [47] Prospective study	5	MRI	Ilium: 3 Scapula: 1 Femur: 1 Humerus: 1	Score: Pre: 5.9 ± 1.3 3 d: 4.7 ± 1.9 7 d: 3.3 ± 2.2 14 d: 2.5 ± 1.3 30 d: 2.9 ± 1.5 60 d: 3.4 ± 1.6 90 d: 0.8 ± 1.1 12 m: 0	Tumor response at 3 m: Reduction in tumor tissue enhancement, and new bone formation in 1 patient.	n/a	2 patients reported improvement in daily activities.	Skin burn: 1 Sonication-related pain: 1, resolved within 2 wk.
Anzidei et al. 2016 [37] Prospective study	23	MRI	Pelvic bone: 12 Scapula: 4 Femur: 2 Humerus: 2 Tibia: 2 Fibula: 1	Score: Pre: 7.09 ± 1.80 1 m: 2.65 ± 1.36 3 m: 1.04 ± 1.91 6 m: 1.09 ± 1.99 Response (IBMCWP): CR: 69.6% PR: 26.1% SD: 4.3%	n/a	n/a	n/a	None reported
Chan et al. 2016 [33] Pilot study	10	MRI	Scapula: 2 Iliac crest: 4 Femur: 1 Hip: 2 Rib: 1	Score: Decreased at 14 d and 30 d follow-up. Response at 14 d (IBMCWP): PR: 37.5% Indeterminate: 50% PD: 12.5% Response at 30 d (IBMCWP): CR: 17% PR: 83%	n/a	n/a	BPI-QoL: All functional scores decreased at 14 d and 30 d follow-up.	None reported
Lee et al. 2017 [38] Retrospective match-pair study with RT	63 (21 HIFU; 42 RT)	MRI	HIFU: Pelvis: 18 Limb: 2 Rib cage: 1 RT: Pelvis: 36 Limb: 4 Rib cage: 2	Score: Pre: HIFU: 6.6 RT: 6.2 1 wk: HIFU: 2.5 RT: 4.8 2 wk: HIFU: 2.1 RT: 3.6 1 m: HIFU: 2.0 RT: 2.8 2 m: HIFU: 1.7 RT: 2.2 3 m HIFU: 2.3 RT: 1.0 Response (IBMCWP): 1 wk: HIFU: 71% RT: 26% 2 wk: HIFU: 76% RT: 50% 1 m: HIFU: 81% RT: 67% 2 m: HIFU: 81% RT: 74% 3 m HIFU: 76% (CR: 43%) RT: 71% (CR: 29%)	Tumor response: Progression observed in 1 HIFU patient and 4 RT patients. Median survival: HIFU: 12.7 m RT: 9.8 m	No significant difference between the mean MEDD change from baseline for the 2 treatment groups at all follow-up time points.	n/a	HIFU: Positioning pain: 3 Sonication pain: 7 Dermatitis: 1 Myositis: 1 RT: Positioning pain: 4 Dermatitis: 3 Diarrhea: 8
Singh et al. 2017 [44] Prospective study	24 (15 primary malignant tumors, 6 bone metastases, 3 osteoid osteomas)	MRI	Femur: 7 Tibia: 7 Pubic rami: 3 Fibula: 3 Humerus: 3 Radius: 1	Score: Pre: 3.04 1 d: 3.17 3 m: 0.7 (only for bone metastasis and osteoid osteoma) Bone metastasis patients remained symptom free with significant decrease in pain scores at 3 m follow-up.	Histopathology for primary malignant tumor: 100% tumor tissue necrosis due to MR-HIFU ablation.	n/a	n/a	Blister: 2 Post procedural pain: 3
Bertrand et al. 2018 [24] Prospective study	17	MRI	Tibia: 2 Femur: 2 Iliac bone: 4 Clavicle: 1 Scapula: 1 Humerus: 1 Ribs: 6	Score: Pre: 7.5 ± 1.3 1 wk: 2.3 ± 1.9 1 m: 1.9 ± 2.0 Response at 1 m (IBMCWP): CR: 37.5% PR: 50.0% PD: 12.5%	n/a	MEDD: Pre: 270.6 (78.2–2293.9) 1 m: 113.75 (44.9–270.0)	n/a	None reported
Chen et al. 2018 [22] Retrospective study	26	MRI	Tibia: 2 Femur: 3 Pelvis: 21	Score: Pre: 6.69 ± 1.49 2 m: 3.96 ± 1.37 4 m: 4.88 ± 1.11 6 m: 5.15 ± 1.08 8 m: 5.34 ± 1.29 10 m: 5.65 ± 1.09 12 m: 5.96 ± 1.14	n/a	n/a	QLQ-BM22: Functional interference: Pre: 89.66 ± 6.54 2 m: 69.83 ± 4.67 4 m: 68.14 ± 5.16 6 m: 66.59 ± 3.93 8 m: 67.19 ± 3.22 10 m: 69.95 ± 4.59 12 m: 72.84 ± 5.13 Psychosocial aspect: Pre: 90.87 ± 4.25 2 m: 59.29 ± 11.86 4 m: 62.66 ± 10.44 6 m: 66.03 ± 5.11 8 m: 69.39 ± 12.79 10 m: 69.39 ± 12.80 12 m: 72.59 ± 12.92	None reported
Einsenberg et al. 2018 [32] Case report	1	MRI	Iliac bone	Increase in local pain during the 1st 3 d followed by improvement in pain response.	Tumor response: No 18F-FDG uptake noted on PET/CT images at 3 m after treatment.	90% reduction in opioid intake at 2 m after treatment.	Patient could ambulate and change positions with no difficulties.	n/a
Harding et al. 2018 [26] Prospective study	20	MRI	Pelvis: 14 Arm: 2 Leg: 1 Rib: 1	Response (IBMCWP): 7 d: 38.9% 14 d: 61.1% 30 d: 53.0% 60 d: 61.5% 90 d: 63.6%	n/a	n/a	QLQ-C15-PAL: Overall QoL: Pre: 49.99 ± 22.04 7 d: 51.85 ± 24.17 14 d: 58.33 ± 22.31 30 d: 50.98 ± 24.62 60 d: 56.41 ± 25.02 90 d: 56.07 ± 27.14 QLQ-BM22: Functional interference: Pre: 45.93 ± 18.08 7 d: 55.09 ± 14.94 14 d: 58.26 ± 15.92 30 d: 52.24 ± 24.64 60 d: 59.80 ± 27.53 90 d: 63.15 ± 23.29 Psychosocial aspect: Pre: 54.63 ± 18.79 7 d: 59.57 ± 16.59 14 d: 57.64 ± 15.96 30 d: 52.94 ± 25.09 60 d: 55.56 ± 17.57 90 d: 55.06 ± 18.17	No treatment-related AE
Wang et al. 2018 [48] Prospective study	21	MRI	Rib cage: 9 Ilium: 8 Humerus: 1 Femur: 1 Sacral vertebra: 1 Pubic bone: 1	Score: Pre: 7.7 ± 1.6 1 wk: 3.6 ± 2.6 1 m: 3.9 ± 3.2 2 m: 4.3 ± 3.3 3 m: 3.7 ± 2.7	n/a	Medication: Increase: 80.0%, 13.3% at 1 m and 2 m due to pain from other organ metastases, 6.7% at 3 m due to tumor progression, and 6.7% at 3 m due to aggravation of bone metastasis pain.	BPI-QoL: Pre: 27.3 ± 20.9 1 wk: 34.1 ± 15.5 1 m: 32.3 ± 19.2 2 m: 31.9 ± 18.9 3 m: 28.8 ± 14.8	1^st^-degree skin burn: 1, improved after symptomatic treatment. Impaired bladder and bowel function: 1, resolved after symptomatic treatment. Low-grade fever: 1, resolved after 1 wk.
Aslani et al. 2019 [49] Pilot study	9	US	Rib: 4 Ulna: 1 Scapula: 2 Iliac crest: 1 Humerus: 1	66.7% had durable pain relief.	n/a	n/a	n/a	n/a
Giles et al. 2019 [50] Prospective study	21 (9 intraosseous group; 12 extraosseous group)	MRI	Intraosseous: Pelvis: 5 Ribs: 2 Humerus: 1 Femur: 1 Extraosseous: Pelvis: 8 Ribs: 1 Humerus: 1 Femur: 1 Sacrum: 1	Score: Intraosseous: Significant reduction in pain scores at 30, 60, and 90 d. Extraosseous: Non-significant reduction in pain scores at 30, 60, and 90 d. Response (IBMCWP): Intraosseous Responder: 67% Extraosseous Responder: 33%	n/a	n/a	n/a	Intraosseous: Post procedural pain: 4 Extraosseous: Temporary numbness of the buttock: 1 Thermal injury of subcutaneous fat: 1
Namba et al. 2019 [25] Retrospective study	11	MRI	n/a	Median score: Pre: 6 (4–8) 3 m: 2 (0–6) Response (OMERACT-OARSI): 1 wk: 60% 1 m: 80% 3 m: 80% Median pressure pain threshold: Pre: 107 kPa (40–432) 3 m: 271 kPa (94–534)		n/a	n/a	n/a
Tsai et al. 2019 [31] Retrospective study	31	MRI	Pelvis: 3 Rib cage: 4	Response at 3 m (IBMCWP): CR: 48.4% PR: 35.5% SD: 12.9% PD: 3.2%	Tumor response at 3 m (modified MDA criteria): CR: 6.5% PR: 61.3% SD: 29% PD: 3.2% 1-year local control rate: 57%.	n/a	n/a	Procedure-related pain: 4, resulting in temporary treatment interruption and additional administration of intravenous morphine administration.
Wang et al. 2019 [18] Prospective study	30	MRI	Ribs: 13 Ilium: 11 Long bones of the extremities: 4 Sacral vertebrae: 2	Score: NRS: Pre: 6.27 ± 1.53 1 wk: 3.69 ± 1.71 1 m: 3.13 ± 1.87 2 m: 2.76 ± 1.53 3 m: 2.18 ± 1.04 VAS: Pre: 6.56 ± 2.38 1 wk: 4.72 ± 2.34 1 m: 3.43 ± 2.16 2 m: 2.29 ± 1.15 3 m: 1.85 ± 0.96	n/a	13 patients had fixed, stable analgesic dosage before HIFU. After HIFU, 6 patients discontinued, 2 patients reduced, and 1 patient had no change in analgesic. At 2 m follow-up, 4 patients discontinued analgesic. 17 patients had no analgesic medication before HIFU, but 1 needed analgesic for failed analgesic effect on unbearable pain and 2 needed analgesic at 3 m follow-up due to other non-treated metastatic bone tumor.	QLQ-C30: Physical function, cognitive function, nausea and vomiting, and degree of pain scores significantly decreased at 1 wk, 1 m, 2 m, and 3 m after HIFU. Total scores: Pre: 16.98 ± 5.38 1 wk: 13.26 ± 3.89 1 m: 12.44 ± 3.20 2 m: 10.80 ± 3.44 3 m: 9.70 ± 2.98	1^st^-degree skin burn: 2, resolved 3 d after symptomatic treatment. Local swelling and numbness: 3, resolved after symptomatic treatment. Low fever: 3, resolved after 1 m.
Drost et al. 2020 [51] Pilot study	9	US	Rib: 4 Ulna: 1 Scapula: 2 Iliac crest: 1 Humerus: 1	Score: Pre: 6.9 10 d: 3.2 Response at 10 d (IBMCWP): CR: 1/9 (11.1%) PR: 8/9 (88.9%)	n/a	MEDD: Pre: 1343 mg/d 10 d: 345 mg/d	QLQ-C15-PAL at 10 d: Scores for questions 1–8 and 11–14 decreased at 10 d follow-up. Scores for nausea remained constant (question 9). Scores for constipation (question 10) increased from 1.3 to 1.7. Overall QoL scores (question 15) increased from an average of 3.8 to 4.6. QLQ-BM22 at 10 d: The average score for 21 questions decreased at 10d follow-up. Scores for pain felt in buttocks (Question 5) increased from 1.3 to 1.6.	Fatigue: 1 Itch: 1 Pain: 3 Redness:1 Sensation: 1, resolved by day 6.
Bongiovanni et al. 2022 [27] Prospective study	12	MRI	Leg: 2 Arm: 1 Scapula: 1 Pelvis: 5 Sacrum: 3	Score: Constant pain: Pre: 3.8 (median) 30 d: 0.7 (mean) Breakthrough pain: Pre: 6.9 (median) 30 d: 1.4 (mean) Response at 30 d: Constant pain: CR: 50% PR: 50% Breakthrough pain: CR: 41.7% PR: 58.3%	Tumor response (MDA criteria): PR: 81.8% Tumor response (PERCIST 1.0): PR: 41.7% SD: 25.0% PD: 33.3%	Median MEDD: Pre: 37.5 mg (0–270 mg) 7 d: 14.3 mg (0–270 mg) 30 d: 7.3 mg (0–180 mg) 90 d: no further increase	Median QLQ-C15-PAL: Physical function: Pre: 40 (26.7–93.3) 1 m: 73.3 (range 26.7–93.3) Emotional function: Pre: 66.7 (0–100) 1 m: 100 (41.7–100)	Grade 2 skin burn: 1 Grade 1 skin edema: 1 Acute pain after sonication: 1
Cabras et al. 2022 [52] First-in-man case report	1	MRI	Arm	Score: Pre: 7 2 d: 8 3 d: 3 3 m: 3	n/a	n/a	n/a	Postprocedural pain
Hsu et al. 2022 [35] Prospective study	20	MRI	Rib: 1 Sternum: 1 Acetabulum: 1 Ilium: 3 Ischium: 1 Sacroiliac joint: 6 Sacrum: 5 Scapula: 2	Response at 3 m (IBMCWP): CR: 80% PR: 20%	Radiographic response at 3 m (modified MDA criteria) ^e^: Overall response rate: 67.7%	n/a	n/a	n/a

Note—MR = magnetic resonance imaging, US = ultrasound, Pre = before HIFU treatment, d = day, wk = week, m = month, CE-T1w = contrast-enhanced T1-weighted, SUV = standard uptake value, HIFU = high intensity focused ultrasound, RT = radiation therapy, CR = complete response, PR = partial response, SD = stable disease, PD = progressive disease, NR = no response, n/a = not available, ^18^F-FDG = ^18^F-fluorodeoxiglucose, PET = positron emission tomography, CT = computed tomography, AE = adverse events, SAE = severe adverse events. Unless otherwise indicated, values represent mean or mean ± standard deviation or mean with range in parenthesis or single value for case report. ^a^ Pain assessment—Pain assessment provides results for pain score and response. Pain scores were assessed using visual analogue scale (VAS) and/or numerical rating scale (NRS) where 0 indicated no pain and 10 indicated severe pain. One study used verbal rating scales for pain score, where 0 indicated no pain, 1 indicated mild pain, 2 indicated moderate pain and 3 indicated severe pain. Pain response was assessed using International Bone Metastases Consensus Working Party (IBMCWP) guidelines, Outcome Measures in Rheumatology Clinical Trials and Osteoarthritis Research Society International (OMERACT-OARSI) and/or pressure pain threshold. ^b^ Tumor response—Tumor response was assessed using MD Anderson (MDA) criteria, modified MDA criteria, World Health Organization (WHO) standard, positron emission tomography (PET) response criteria in solid tumors (PERCIST 1.0), ^18^F-FDG PET/CT and/or CE-T1w MRI. ^c^ Pain medication or morphine equivalent daily dose (MEDD) was used to assess changes in analgesic intake. ^d^ Quality of Life (QoL)—QoL after treatments was assessed using brief pain inventory (BPI) QoL (BPI-QoL) questionnaire, European Organization for Research and Treatment of Cancer (EORTC) Quality of Life Questionnaire Core 15 Palliative Care (QLQ-C15-PAL), EORTC QoL Questionnaires for Patients with Bone Metastases 22 (QLQ-BM22) and/or EORTC QLQ-C30. QLQ-BM22 has four sub-analyses: painful sites, painful characteristics, functional interference and psychosocial aspects. As pain response has already been reported using the analyses under “Pain assessment”, only functional interference-related and psychosocial aspect-related results were added to the table. ^e^ Due to contradictory information between the Results and Discussion sections in the publication, only data from the Results section are listed.

## 4. Discussion

Findings from all studies show that HIFU provides rapid and lasting pain palliation in patients suffering from painful bone metastases or primary tumors. Typically, the onset of pain relief is between 3 days and 2 weeks after treatment, and many times, pain medication could be reduced or stopped. Until now, HIFU was used in most bone metastases-related studies as a second line treatment option in patients with recurring pain after radiotherapy treatment. Studies in primary tumors as well as bone metastases show that HIFU provides local tumor control with normalization of the bone structure evidenced by remineralization visible in CT or metabolic changes visualized with PET and SPECT imaging. Furthermore, HIFU is a very safe method with mainly transient treatment-related effects such as occasional skin burns or a pain flair shortly after treatment, all resolving within a few days after therapy, making it a very patient-friendly treatment option.

Compared with current treatment options for bone metastasis or primary malignant bone tumor, such as RT, surgery with or without chemotherapy, RFA and/or MW ablation, HIFU has the advantage of being a safe and effective method, especially in providing rapid pain relief. Being non-invasive and using non-ionizing ultrasound waves, HIFU can be repeated without dose limitation as in the case of RT. An early economic modeling study demonstrated that adding MR-HIFU to RT regimes, independent of the sequence of treatment, resulted in better quality-adjusted life years (QALYs) and more durable pain response, compared with RT alone for pain palliation of bone metastasis. Furthermore, MR-HIFU has a 52% probability of being cost-effective compared with RT, according to probabilistic sensitivity analysis in the treatment of bone metastasis [53]. On the other hand, the shortcomings of HIFU include its long procedural time and the fact that it is limited to lesions that have unobstructed acoustic pathways, i.e., lesions that can be accessed and treated by HIFU and can be reached by a transducer. Furthermore, lesions that are in the skull or spine, with the exception of the posterior elements below the level of the conus medullaris, are currently excluded from HIFU [13].

To address the current drawbacks of HIFU, future developments could focus on improving workflow efficiency and treatment efficacy. For example, it is essential to understand the extent of tissue damage in the near-field and beyond the cortical bone for different combinations of HIFU parameters, such as sonication duration, power, and frequency. These results can be used to develop a (semi)-automatic treatment planning tool that comes with thermal modeling capabilities which will suggest an optimum treatment plan with respect to the defined target location and aims, i.e., pain palliation and/or tumor control, as well as simulate temperature increase and tissue necrosis for the selected HIFU parameters. Based on the simulation results, clinicians can then design a personalized treatment strategy to ensure optimum treatment outcomes while minimizing the treatment duration and/or side effects. Besides thermal ablation, HIFU can also induce mechanical ablation, known as histotripsy [54,55]. The feasibility of performing histotripsy in bone tumors has been demonstrated in excised canine osteosarcoma tumors [56]. Additional studies should be conducted to understand the biological and mechanical effects of histotripsy on bone tumors. Continuous research and development of HIFU for treatment of malignant bone tumors will hopefully allow us to include patients who were previously not eligible for HIFU, thereby increasing clinical use and benefiting a larger patient population.

## 5. Conclusions

HIFU has shown its value in many studies and has emerged as a competitive option for radiotherapy and other thermal ablation techniques. The key to success is surely proper patient selection, considering stage of disease and treatment objectives but also the treatability of the target lesion using HIFU. Future randomized prospective studies have to be performed to provide medical evidence for HIFU as a first line treatment option for pain palliation or the management of local bone lesions.

## Figures and Tables

**Table 2:** A summary of clinical studies on HIFU treatment of primary malignant tumors.

Study	Patient Number	Imaging Guidance	Lesion Location	Follow-up	Pain Assessment ^a^ and Quality of Life ^b^	Tumor Response ^c^ and Survival
Chen et al. 2002 [40] Retrospective study	44	US	Distal femur: 20 Proximal and middle: 7 Proximal tibia: 6 Tibiofibular shaft: 1 Fibula shaft: 2 Proximal humerus: 1 Ulna: 2 Ilium: 3 Public bone: 1 Multiple foci in different bones: 1	MRI, CT and or SPECT (^99m^Tc-MDP) at 7–14 d, 3 m, 9 m, 15 m, 21 m, 27 m, 33 m	Enneking system: 81.8% ≥ 15 points (average 21.5 points)	Tumor response: 7–14 d: radioactive cold tumor Survival: Overall: 84.1% Tumor-free survival: 68.2% Survival with tumor: 15.9% Local recurrence: 9.1% Stage IIb (n = 34): Disease-free: 30 (mean survival 21.7 m) Recurrence: 2 Passed away due to brain and lung metastasis: 2 Stage IIIb (n = 10): Survived with tumor: 4 Survived with tumor & recurrence: 1 Pass away due to lung metastasis: 5
Li et al. 2009 [41] Retrospective study	7	US	Distal femur: 4 Distal humerus: 1 Proximal humerus: 1 Proximal tibia: 1	MRI, SPECT (^99m^Tc-MDP) at 1,3 m	Score: (VRS *) Pre: Severe pain: 2 Moderate pain: 2 Mild pain: 3 1 & 3 m: All patients are pain free after treatment. No further pain medication needed after HIFU.	Tumor response: CP: 42.9 % PR: 42.9 % PD: 14.2 % Survival Median survival: 68 m 5 years survival: 71.4%
Li et al. 2010 [23] Retrospective study	25 13 patients with primary osteosarcoma 12 patients with bone metastasis (not further considered in this table)	US	Distal femur: 4, Distal humerus: 1 Proximal tibia: 1 Proximal humerus: 1 Left ilium: 1 Right ilium: 2 Right scapula: 1 Right rib: 1 Left pubis: 1	MRI, PET (^18^F-FDG), SPECT (^99m^Tc-MDP), MRI, CT 4–6 wks after HIFU.	Score: (VRS *) Pre: Severe pain: 2 Moderate pain: 7 Mild pain: 4 Mean: 1.85 ± 0.69 4–6 wks Mean: 0.12 ± 0.33	Tumor response (WHO): CR: 46.2% PR: 38.5% MR: 7.8% SD: 0% PD: 7.8% RR: 84.6% Survival: 1, 2, 3, 5 years survival was 100.0%, 84.6%, 69.2% and 38.5%.
Chen et al. 2010 [42] Retrospective study	80	US	Distal femur: 33 Proximal tibia: 14 Middle tibia: 3 Distal tibia: 1 Fibula shaft: 2 Tibiofibular shaft: 1 Proximal humerus: 4 Ulna: 1 Rib: 3 Pelvis, multi foci in different bones: 1	CT, MRI (1.5 T), SPECT (^99m^Tc-MDP) at 14d	n/a	Tumor response: 100% tumor ablation in 69 patients. >50% ablation in 11 patients. Overall survival rates At year 1, 2, 3, 4, 5 were 89.8%, 72.3%, 60.5%, 50.5%, and 50.5%.
Wang et al. 2013 [43] Retrospective study	11	US	Ilium: 8 Ischium: 1 Both ilium and ischium: 2	CE-MRI at 1, 3, 6 m and every 6–7 m thereafter	Pre: Mild to moderate pain in all patients; 6 patients received oral analgesics. Post: Transient pain after treatment resolved within 3 d. Patients were pain free thereafter, analgesics were discontinued.	Tumor response: 4 patients treated with curative intent: CR: 100% (complete ablation) 7 patients with palliative treatment: PR: 79.1 ± 8.7 %
Singh et al. 2017 [44] Retrospective study	24 (15 primary malignant tumors, 6 bone metastases, 3 osteoid osteomas)	MRI	Femur: 7 Tibia: 7 Pubic rami: 3 Fibula: 3 Humerus: 3 Radius: 3	Primary tumors were resected 14 d after HIFU for histology.	Score: Pre: 3.04 1 d: 3.17 3 m: 0.7 (only for bone metastasis and osteoid osteoma) Bone metastasis patients remained symptom free with significant decrease in pain scores at 3 m follow-up.	Tumor response: 100% necrosis in areas treated with MR-HIFU
Wang C. 2019 [45] Prospective two-arm study	72	US	Control Group (CG)/Observation Group(OG): Upper tibia: 19/18 Lower femur: 6/5 Upper femur: 3/2 Humerus: 2/3 Upper ulna: 1/2 Upper fibula: 1/2 Scapula: 1/3 Ilium: 1/1	Control group (CG): adriamycin (36) vs. Bbservation group (OG): adriamycin + HIFU (36) Endpoints: Treatment efficacy: overall response, disease control, survival.	Quality of Life: Post: Significantly improved limb function and psychological behavior for OG compared with CG.	Overall response: OG group 88.9% vs. CG: 66.7 % Disease control rate: OG: 94.4% vs. CG: 75 % Survival OG/CG: 1 year: OG: 83.3% vs. CG: 75%, no difference 2 years: OG: 69.4% vs. CG: 52.8 % 3 years: OG: 38.9% vs. CG: 22.2%

Note—MR = magnetic resonance imaging, US = ultrasound, Pre = Before HIFU treatment, d = day, wk = week, m = month, HIFU = high intensity focused ultrasound, CR = complete response, PR = partial response, SD = stable disease, PD = progressive disease, MR = moderate response, RR = total effective rate, NR = no response, n/a = not available, ^18^F-FDG = ^18^F-fluorodeoxiglucose, PET = positron emission tomography, CT = computed tomography, SPECT = single-photon emission computed tomography, ^99m^Tc-MDP = Technetium 99 m-methyl diphosphonate, AE = adverse events, SAE = severe adverse events. Unless otherwise indicated, values represent mean or mean ± standard deviation or mean with range in parenthesis or single value for case report. ^a^ Pain assessment—Pain assessment provides results for pain score and response. Pain scores were assessed using visual analogue scale (VAS) and/or numerical rating scale (NRS) where 0 indicated no pain, while 10 indicated severe pain. One study used verbal rating scales (VRS) * for pain score, where 0 indicated no pain, 1 indicated mild pain, 2 indicated moderate pain and 3 indicated severe pain. ^b^ Quality of Life (QoL) was assessed using the Enneking system, wherein <15 points = poor function and self-care not possible, 15–20 points = normal function with possible self-care, ≥21 points = patients are able to lead a normal life. ^c^ Tumor response—World Health Organization (WHO) standard, ^18^F-FDG PET/CT, ^99m^Tc-MDP SPECT/CT and/or MRI.

## References

[1] Mundy G.R. (2002). Metastasis to Bone: Causes, Consequences and Therapeutic Opportunities. Nat. Rev. Cancer.

[2] Siegel R.L., Miller K.D., Fuchs H.E., Jemal A. (2022). Cancer Statistics, 2022. CA Cancer J. Clin..

[3] Mantyh P. (2013). Bone Cancer Pain: Causes, Consequences, and Therapeutic Opportunities. Proc. Pain.

[4] Lutz S., Berk L., Chang E., Chow E., Hahn C., Hoskin P., Howell D., Konski A., Kachnic L., Lo S. (2011). Palliative Radiotherapy for Bone Metastases: An ASTRO Evidence-Based Guideline. Int. J. Radiat. Oncol. Biol. Phys..

[5] Ringe K., Panzica M., von Falck C. (2016). Thermoablation of Bone Tumors. RöFo Fortschr. Geb. Der Röntgenstrahlen Bildgeb. Verfahr..

[6] Bazzocchi A., Aparisi Gómez M.P., Taninokuchi Tomassoni M., Napoli A., Filippiadis D., Guglielmi G. (2022). Musculoskeletal Oncology and Thermal Ablation: The Current and Emerging Role of Interventional Radiology. Skelet. Radiol..

[7] Ter Haar G., Coussios C. (2007). High Intensity Focused Ultrasound: Physical Principles and Devices. Int. J. Hyperth..

[8] Quesson B., De Zwart J.A., Moonen C.T.W. (2000). Magnetic Resonance Temperature Imaging for Guidance of Thermotherapy. J. Magn. Reson. Imaging.

[9] Siedek F., Yeo S.Y., Heijman E., Grinstein O., Bratke G., Heneweer C., Puesken M., Persigehl T., Maintz D., Grull H. (2019). Magnetic Resonance-Guided High-Intensity Focused Ultrasound (MR-HIFU): Technical Background and Overview of Current Clinical Applications (Part 1). RoFo Fortschr. Geb. Rontgenstrahlen Bildgeb. Verfahr..

[10] Siedek F., Yeo S.Y., Heijman E., Grinstein O., Bratke G., Heneweer C., Puesken M., Persigehl T., Maintz D., Grüll H. (2019). Magnetic Resonance-Guided High-Intensity Focused Ultrasound (MR-HIFU): Overview of Emerging Applications (Part 2). RoFo Fortschr. Geb. Rontgenstrahlen Bildgeb. Verfahr..

[11] Focused Ultrasound Foundation State of the Field Report 2022. https://cdn.fusfoundation.org/2022/11/01111233/Focused-Ultrasound-Foundation_State-of-the-Field-Report-2022_Nov1.pdf.

[12] Ten Eikelder H.M.M., Bosnacki D., Elevelt A., Donato K., Di Tullio A., Breuner B.J.T., van Wijk J.H., Yeo S.Y., Grüll H. (2016). Modeling the Temperature Evolution of Bone under High Intensity Focused Ultrasound. Phys. Med. Biol..

[13] Huisman M., Ter Haar G., Napoli A., Hananel A., Ghanouni P., Lövey G., Nijenhuis R.J., van den Bosch M.A.A.J., Rieke V., Majumdar S. (2015). International Consensus on Use of Focused Ultrasound for Painful Bone Metastases: Current Status and Future Directions. Int. J. Hyperth..

[14] Napoli A., Anzidei M., Marincola B.C., Brachetti G., Noce V., Boni F., Bertaccini L., Passariello R., Catalano C. (2013). MR Imaging–Guided Focused Ultrasound for Treatment of Bone Metastasis. RadioGraphics.

[15] Hurwitz M.D., Ghanouni P., Kanaev S.V., Iozeffi D., Gianfelice D., Fennessy F.M., Kuten A., Meyer J.E., LeBlang S.D., Roberts A. (2014). Magnetic Resonance-Guided Focused Ultrasound for Patients with Painful Bone Metastases: Phase III Trial Results. J. Natl. Cancer Inst..

[16] Catane R., Beck A., Inbar Y., Rabin T., Shabshin N., Hengst S., Pfeffer R.M., Hanannel A., Dogadkin O., Liberman B. (2007). MR-Guided Focused Ultrasound Surgery (MRgFUS) for the Palliation of Pain in Patients with Bone Metastases—Preliminary Clinical Experience. Ann. Oncol..

[17] Gianfelice D., Gupta C., Kucharczyk W., Bret P., Havill D., Clemons M. (2008). Palliative Treatment of Painful Bone Metastases with MR Imaging--Guided Focused Ultrasound. Radiology.

[18] Wang B., Li J., Wei X. (2019). Short-Term Efficacy and Safety of MR-Guided Focused Ultrasound Surgery for Analgesia in Children with Metastatic Bone Tumors. Oncol. Lett..

[19] Han X., Huang R., Meng T., Yin H., Song D. (2021). The Roles of Magnetic Resonance-Guided Focused Ultrasound in Pain Relief in Patients With Bone Metastases: A Systemic Review and Meta-Analysis. Front. Oncol..

[20] Baal J.D., Chen W.C., Baal U., Wagle S., Baal J.H., Link T.M., Bucknor M.D. (2021). Efficacy and Safety of Magnetic Resonance-Guided Focused Ultrasound for the Treatment of Painful Bone Metastases: A Systematic Review and Meta-Analysis. Skelet. Radiol..

[21] Candiano G., Russo G., Stefano A., Marino L., Ganguzza F., Vaccari A., Tripoli V., Galluzzo A., Pulizzi S., Messana D. (2012). Metabolic Changes after MRgFUS Treatment of a Bone Metastasis Using PET/CT: A Case Report. Proceedings of the 12th International Symposium on Therapeutic Ultrasound.

[22] Chen Z.-q., Wang C.-r., Ma X.-j., Sun W., Shen J.-k., Sun M.-x., Fu Z.-z., Hua Y.-q., Cai Z. (2018). dong Evaluation of Quality of Life Using EORTC QLQ-BM22 in Patients with Bone Metastases after Treatment with Magnetic Resonance Guided Focused Ultrasound. Orthop. Surg..

[23] Li C., Zhang W., Fan W., Huang J., Zhang F., Wu P. (2010). Noninvasive Treatment of Malignant Bone Tumors Using High-Intensity Focused Ultrasound. Cancer.

[24] Bertrand A.-S., Iannessi A., Natale R., Beaumont H., Patriti S., Xiong-Ying J., Baudin G., Thyss A. (2018). Focused Ultrasound for the Treatment of Bone Metastases: Effectiveness and Feasibility. J. Ther. Ultrasound.

[25] Namba H., Kawasaki M., Izumi M., Ushida T., Takemasa R., Ikeuchi M. (2019). Effects of MRgFUS Treatment on Musculoskeletal Pain: Comparison between Bone Metastasis and Chronic Knee/Lumbar Osteoarthritis. Pain Res. Manag..

[26] Harding D., Giles S.L., Brown M.R.D., ter Haar G.R., van den Bosch M., Bartels L.W., Kim Y.-S., Deppe M., deSouza N.M. (2018). Evaluation of Quality of Life Outcomes Following Palliative Treatment of Bone Metastases with Magnetic Resonance-Guided High Intensity Focused Ultrasound: An International Multicentre Study. Clin. Oncol..

[27] Bongiovanni A., Foca F., Oboldi D., Diano D., Bazzocchi A., Fabbri L., Mercatali L., Vanni S., Maltoni M., Bianchini D. (2022). 3-T Magnetic Resonance–Guided High-Intensity Focused Ultrasound (3 T-MR-HIFU) for the Treatment of Pain from Bone Metastases of Solid Tumors. Support. Care Cancer.

[28] Gu J., Wang H., Tang N., Hua Y., Yang H., Qiu Y., Ge R., Zhou Y., Wang W., Zhang G. (2015). Magnetic Resonance Guided Focused Ultrasound Surgery for Pain Palliation of Bone Metastases: Early Experience of Clinical Application in China. Zhonghua Yi Xue Za Zhi.

[29] Yeo S.Y., Elevelt A., Donato K., van Rietbergen B., ter Hoeve N.D., van Diest P.J., Grüll H. (2015). Bone Metastasis Treatment Using Magnetic Resonance-Guided High Intensity Focused Ultrasound. Bone.

[30] Napoli A., Anzidei M., Marincola B.C., Brachetti G., Ciolina F., Cartocci G., Marsecano C., Zaccagna F., Marchetti L., Cortesi E. (2013). Primary Pain Palliation and Local Tumor Control in Bone Metastases Treated with Magnetic Resonance-Guided Focused Ultrasound. Investig. Radiol..

[31] Tsai Y.C., Lee H.L., Kuo C.C., Chen C.Y., Hsieh K.L.C., Wu M.H., Wen Y.C., Yu H.W., Hsu F.C., Tsai J.T. (2019). Prognostic and Predictive Factors for Clinical and Radiographic Responses in Patients with Painful Bone Metastasis Treated with Magnetic Resonance-Guided Focused Ultrasound Surgery. Int. J. Hyperth..

[32] Eisenberg E., Shay L., Keidar Z., Amit A., Militianu D. (2018). Magnetic Resonance-Guided Focused Ultrasound Surgery for Bone Metastasis: From Pain Palliation to Biological Ablation?. J. Pain Symptom Manag..

[33] Chan M., Dennis K., Huang Y., Mougenot C., Chow E., DeAngelis C., Coccagna J., Sahgal A., Hynynen K., Czarnota G. (2017). Magnetic Resonance–Guided High-Intensity-Focused Ultrasound for Palliation of Painful Skeletal Metastases: A Pilot Study. Technol. Cancer Res. Treat..

[34] Bushehri A., Czarnota G., Zhang L., Hynynen K., Huang Y., Chan M., Chu W., Dennis K., Mougenot C., Coccagna J. (2017). Urinary Cytokines/Chemokines after Magnetic Resonance-Guided High Intensity Focused Ultrasound for Palliative Treatment of Painful Bone Metastases. Ann. Palliat. Med..

[35] Hsu F.C., Lee H.L., Chen Y.J., Shen Y.A., Tsai Y.C., Wu M.H., Kuo C.C., Lu L.S., Der Yeh S., Huang W.S. (2022). A Few-Shot Learning Approach Assists in the Prognosis Prediction of Magnetic Resonance-Guided Focused Ultrasound for the Local Control of Bone Metastatic Lesions. Cancers.

[36] Liberman B., Gianfelice D., Inbar Y., Beck A., Rabin T., Shabshin N., Chander G., Hengst S., Pfeffer R., Chechick A. (2009). Pain Palliation in Patients with Bone Metastases Using MR-Guided Focused Ultrasound Surgery: A Multicenter Study. Ann. Surg. Oncol..

[37] Anzidei M., Napoli A., Sacconi B., Boni F., Noce V., Di Martino M., Saba L., Catalano C. (2016). Magnetic Resonance-Guided Focused Ultrasound for the Treatment of Painful Bone Metastases: Role of Apparent Diffusion Coefficient (ADC) and Dynamic Contrast Enhanced (DCE) MRI in the Assessment of Clinical Outcome. Radiol. Med..

[38] Lee H.-L., Kuo C.-C., Tsai J.-T., Chen C.-Y., Wu M.-H., Chiou J.-F. (2017). Magnetic Resonance-Guided Focused Ultrasound Versus Conventional Radiation Therapy for Painful Bone Metastasis: A Matched-Pair Study. J. Bone Jt. Surg..

[39] Bartels M.M.T.J., Verpalen I.M., Ferrer C.J., Slotman D.J., Phernambucq E.C.J., Verhoeff J.J.C., Eppinga W.S.C., Braat M.N.G.J.A., van den Hoed R.D., van ’t Veer-Ten Kate M. (2021). Combining Radiotherapy and Focused Ultrasound for Pain Palliation of Cancer Induced Bone Pain; a Stage I/IIa Study According to the IDEAL Framework. Clin. Transl. Radiat. Oncol..

[40] Chen W., Wang Z., Wu F., Zhu H., Zou J., Bai J., Li K., Xie F. (2002). High Intensity Focused Ultrasound in the Treatment of Primary Malignant Bone Tumor. Zhonghua Zhong Liu Za Zhi.

[41] Li C., Wu P., Zhang L., Fan W., Huang J., Zhang F. (2009). Osteosarcoma: Limb Salvaging Treatment by Ultrasonographically Guided High-Intensity Focused Ultrasound. Cancer Biol. Ther..

[42] Chen W., Zhu H., Zhang L., Li K., Su H., Jin C., Zhou K., Bai J., Wu F., Wang Z. (2010). Primary Bone Malignancy: Effective Treatment with High-Intensity Focused Ultrasound Ablation. Radiology.

[43] Wang Y., Wang W., Tang J. (2013). Primary Malignant Tumours of the Bony Pelvis: US-Guided High Intensity Focused Ultrasound Ablation. Int. J. Hyperth..

[44] Singh V.A., Shah S.U., Yasin N.F., Abdullah B.J.J. (2017). Magnetic Resonance Guided Focused Ultrasound for Treatment of Bone Tumors. J. Orthop. Surg..

[45] Wang C. (2019). Therapeutic Effects of Adriamycin Combined with High-Intensity Focused Ultrasound on Osteosarcoma. J. Balk. Union Oncol..

[46] Huisman M., Lam M.K., Bartels L.W., Nijenhuis R.J., Moonen C.T., Knuttel F.M., Verkooijen H.M., van Vulpen M., van den Bosch M.A. (2014). Feasibility of Volumetric MRI-Guided High Intensity Focused Ultrasound (MR-HIFU) for Painful Bone Metastases. J. Ther. Ultrasound.

[47] Joo B., Park M.-S., Lee S.H., Choi H.J., Lim S.T., Rha S.Y., Rachmilevitch I., Lee Y.H., Suh J.-S. (2015). Pain Palliation in Patients with Bone Metastases Using Magnetic Resonance-Guided Focused Ultrasound with Conformal Bone System: A Preliminary Report. Yonsei Med. J..

[48] Wang S., Sun Z., Xin C., Du C., Xu L., Gu Y., Li W., Peng W. (2018). Magnetic Resonance-Guided Focused Ultrasound Surgery for Pain Palliation of Bone Metastases: Preliminary Study on the Short-Term Efficacy and Safety. J. Interv. Radiol..

[49] Aslani P., Drost L., Huang Y., Lucht B.B.C., Wong E., Czarnota G., Yee C., Wan B.A., Ganesh V., Gunaseelan S.T. (2020). Thermal Therapy with a Fully Electronically Steerable HIFU Phased Array Using Ultrasound Guidance and Local Harmonic Motion Monitoring. IEEE Trans. Biomed. Eng..

[50] Giles S.L., Brown M.R.D., Rivens I., Deppe M., Huisman M., Kim Y.-S., Farquhar-Smith P., Williams J.E., ter Haar G.R., deSouza N.M. (2019). Comparison of Imaging Changes and Pain Responses in Patients with Intra- or Extraosseous Bone Metastases Treated Palliatively with Magnetic Resonance-Guided High-Intensity–Focused Ultrasound. J. Vasc. Interv. Radiol..

[51] Drost L., Hynynen K., Huang Y., Lucht B., Wong E., Czarnota G., Yee C., Wan B.A., Ganesh V., Chow E. (2020). Ultrasound-Guided Focused Ultrasound Treatment for Painful Bone Metastases: A Pilot Study. Ultrasound Med. Biol..

[52] Cabras P., Auloge P., Bing F., Rao P.P., Hoarau S., Dumont E., Durand A., Maurin B., Wach B., Cuvillon L. (2022). A New Versatile MR-Guided High-Intensity Focused Ultrasound (HIFU) Device for the Treatment of Musculoskeletal Tumors. Sci. Rep..

[53] Simões Corrêa Galendi J., Yeo S.Y., Grüll H., Bratke G., Akuamoa-Boateng D., Baues C., Bos C., Verkooijen H.M., Shukri A., Stock S. (2022). Early Economic Modeling of Magnetic Resonance Image-Guided High Intensity Focused Ultrasound Compared to Radiotherapy for Pain Palliation of Bone Metastases. Front. Oncol..

[54] Khokhlova V.A., Fowlkes J.B., Roberts W.W., Schade G.R., Xu Z., Khokhlova T.D., Hall T.L., Maxwell A.D., Wang Y.N., Cain C.A. (2015). Histotripsy Methods in Mechanical Disintegration of Tissue: Towards Clinical Applications. Int. J. Hyperth..

[55] Hoogenboom M., Eikelenboom D., den Brok M.H., Heerschap A., Fütterer J.J., Adema G.J. (2015). Mechanical High-Intensity Focused Ultrasound Destruction of Soft Tissue: Working Mechanisms and Physiologic Effects. Ultrasound Med. Biol..

[56] Arnold L., Hendricks-Wenger A., Coutermarsh-Ott S., Gannon J., Hay A.N., Dervisis N., Klahn S., Allen I.C., Tuohy J., Vlaisavljevich E. (2021). Histotripsy Ablation of Bone Tumors: Feasibility Study in Excised Canine Osteosarcoma Tumors. Ultrasound Med. Biol..

